# Efficient User-Serving Scheme in the User-Centric Cell-Free Massive MIMO System

**DOI:** 10.3390/s22103794

**Published:** 2022-05-17

**Authors:** Ji-Hye Oh, Beom-Sik Shin, Min-A Kim, Young-Hwan You, Duck-Dong Hwang, Hyoung-Kyu Song

**Affiliations:** 1 Department of Information and Communication Engineering, Sejong University, Seoul 05006, Korea; wlgp5329@naver.com (J.-H.O.); hidex9852@yahoo.com (B.-S.S.); happy990927@naver.com (M.-A.K.); 2 Department of Convergence Engineering for Intelligent Drone, Sejong University, Seoul 05006, Korea; yhyou@sejong.ac.kr; 3 Department of Computer Engineering, Sejong University, Seoul 05006, Korea; 4 Department of Electronics and Communication Engineering, Sejong University, Seoul 05006, Korea; duckdonh@sejong.ac.kr

**Keywords:** 5G, cell-free massive MIMO, user-centric cell-free massive MIMO, unserved users

## Abstract

A cell-free massive multiple input multiple output (MIMO) system is an attractive network model that is in the spotlight in 5G and future communication systems. Despite numerous advantages, the cell-free massive MIMO system has a problem in that it is difficult to operate in reality due to its vast amount of calculation. The user-centric cell-free massive MIMO model has a more feasible and scalable benefit than the cell-free massive MIMO model. However, this model has the disadvantage that as the number of users in the area increases, there are users who do not receive the service. In this paper, the proposed scheme creates connections for unserved users under a user-centric scheme without additional access point (AP) installation and disconnection for existing users. A downlink user-centric cell-free massive MIMO system model in which the APs are connected to the central processing unit (CPU) and the APs and users are geographically distributed is considered. First, the downlink spectral efficiency formula is derived and applied to the user-centric cell-free massive MIMO system. Then, the proposed scheme and power control algorithm are applied to the derived formula. The simulation results show that the unserved users within the area disappear by using the proposed scheme, while the bit error rate (BER) performance and sum rate improve compared to the existing scheme. In addition, it is shown that the proposed scheme works well even with a very large number of users in the area, and a significant service performance improvement for the worst 10% of users and the overall improvement of per-user throughput for the bottom 70% of users are ensured.

## 1. Introduction

A massive MIMO system is a technology that can achieve high throughput by using a large antenna array, and it has attracted attention as a recent 5G system and a promising future technology [1,2,3]. Because of the structural advantages of massive MIMO, recently, much research has been conducted based on that structure [4,5]. The cell-free massive MIMO system also uses multiple antennas that are called access points (APs) as a massive MIMO, and the APs are geographically distributed [6,7,8]. This structure can also achieve the advantages of the previous massive MIMO such as channel hardening and favorable propagation [9]. Due to this network structure, cell-free massive MIMO is robust against the influence of the surrounding environment and shadow fading; so, it can provide uniform spectral efficiency for all users in the area [8,10,11].

The early version of this model was that all APs would serve all users, as in [8]. Since the model in [8] requires a very high amount of computational complexity and fronthaul requirements, which increase in proportion to the number of users [11,12,13], the initial cell-free model was non-scalable and difficult to apply in reality [14,15].

To address this disadvantage, the user-centric cell-free massive MIMO concept appeared [16,17]. The user-centric model was proposed in [18,19]. In this model, all APs do not serve all users; so, it requires much lower computational complexity and fronthaul requirements compared to the previous cell-free massive MIMO system [18]. Therefore, it is a feasible and scalable approach in terms of actual implementation and computational complexity even as the number of users increases. In a 5G communication system, power consumption and energy efficiency should be carefully considered in addition to conventional indicators such as error probability and sum rate [13]. The original cell-free massive MIMO model in [8] has limitations in terms of power consumption and energy efficiency, since the complexity increases proportionally as the number of users increases. However, the user-centric cell-free massive MIMO model adopted in this paper can reasonably maintain the complexity of the entire system even if the number of users in the area increases, requiring lower power consumption and ensuring energy efficiency. Basically, both the cell-free massive MIMO system model and the user-centric cell-free massive MIMO model assume that the number of antennas is much larger than the number of users of the massive MIMO [8]. However, in a realistic situation, the number of users can also exist in a non-negligible amount, and when there are many users under the user-centric scheme, there is a high possibility that users who can not receive the service exist. To solve this problem, a user-serving scheme for connecting unserved users was proposed [20]. However, this scheme had the following limitations: it did not consider the case in which the number of APs is similar to the number of users as in  [21]. Furthermore, when the number of APs and users is almost the same, there is a possibility that other unserved users may occur. In addition, this scheme used a method of simply selecting the closest AP to an unserved user as [22]. In this case, that can damage the data rate of users that are already served in order to serve the unserved user.

Therefore, in this paper, when many users exist in the user-centric cell-free massive MIMO system model, the proposed scheme can efficiently serve the unserved users while maintaining service for existing users. By applying the proposed scheme, it is possible to serve new users at the same time without significantly impairing the throughput of existing users. The main contributions of this paper are outlined as follows:

- We first derive the downlink spectral efficiency in the user-centric cell-free massive MIMO system, which considers the minimum mean square error (MMSE) estimator channel estimation and equal gain power control [8,19].
- In order to keep the computational complexity and fronthaul requirements to a realistic level, conjugate beamforming (CB) is used for precoding, and the equal gain algorithm is used for power control [8,13]. Each AP performs channel estimation locally, and the channel model used in this paper is assumed to be flat fading [10,11]. In addition, all APs support users as the number of installed antennas.
- The proposed scheme is derived from the distribution of per-user throughput. It is shown that the majority of per-user throughput is obtained from only a few APs. Based on this observation, the unserved users can be served while existing user data rates are maintained. By applying the proposed scheme, it is possible to obtain an improved per-user throughput through the cumulative distribution function (CDF) for the worst 10% of users compared to the previous scheme in [20] with many users, and also to achieve an improved sum throughput and BER performance.
- In this paper, the sum rate performance simulation is conducted in several situations for comparison . For sensitivity analysis, the situation where perfect channel state information (CSI) or channel estimate is applied is compared, and a simulation is performed on the effect of the limitation on the number of pilot sequences.

This paper is organized as follows: In Section 2, the network model and downlink spectral efficiency are described. In Section 3, the observation of distribution for per-user throughput is shown. Section 4 discusses the proposed scheme algorithm and the complexity. The power control and pilot assignment method are also explained. Section 5 provides the simulation results, and Section 6 summarizes the paper. Table 1 describes the notations and symbols used in this paper.

## 2. Network Model and Spectral Efficiency

**Figure 1:**
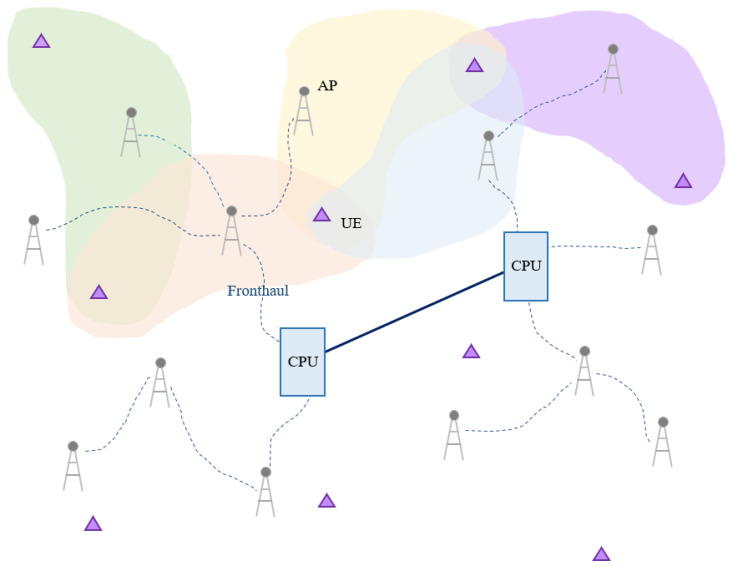
Network model of user-centric cell-free massive MIMO system.

The model of the user-centric cell-free massive MIMO system is shown in Figure 1. This paper considers L APs equipped with N antennas and K single antenna user equipments (UEs). All APs are connected to the CPU through the fronthaul, and it is assumed that APs and UEs operate in time division duplexing (TDD) as a massive MIMO [1]. Each AP does not serve all users but only a few users. In this network model, each coherence block is represented by $\tau_{c}$ and is divided into three phases

(1) $$g_{kl}∼N_{C}\left(0,1\right).$$

In the $h_{kl}$, $\sqrt{\beta_{mk}}$ represents the large scale fading that consists of pathloss and shadow fading, while $g_{mk}$ represents the small-scale fading. For all *l* = 1, …, L, *k* = 1, …, K, $g_{mk}$ is assumed to be independent and identically distributed (i.i.d.) random variables [23,24]. This paper defines M=LN; then, the collective channel of *k* can be represented as $h_{kl}={\left[h_{k1}^{T},h_{k2}^{T},\ldots ,h_{kl}^{T}\right]}^{T}\in C^{M}$. In this paper, $q_{k}$ denotes the symbol for *k* user, and it satisfies $E\left\{|q_{k}{|}^{2}\right\}=1.$ All of the symbols are mutually independent, and all channel coefficients and noise are independent of the symbols. In addition, the matrix α, which indicates the connection between APs and UEs, is defined. If AP *l* and user *k* are connected, $\alpha_{kl}=1$; otherwise, it has a value of 0. This matrix makes the difference between the original cell-free system and the user-centric system.

### 2.1. Uplink Pilot Transmission and Channel Estimation

When the $\sqrt{\tau_{p}}\varphi_{k}\in C^{\tau_{p}\times 1}$ is defined, the $\tau_{p}$ is a pilot transmission length of coherence block $\tau_{c}$, and $\left|\right|\varphi_{k}|{|}^{2}=1$ is a pilot sequence for user *k*. Then, the received pilot signal from the AP *l* to user *k* is given by

(2) $$y_{p,l}=\sqrt{\tau_{p}\rho_{p}}\sum_{k=1}^{K}h_{lk}\varphi_{k}+\omega_{p,l},$$

where $\rho_{p}$ is the power of the pilot sequence, and $\omega_{p,l}$ is a vector of additive noise at the AP *l* which has i.i.d. $N_{C}\left(0,1\right)$. Then, the AP *l* estimates the channel $h_{kl}$ locally based on the received pilot signal (2) by using the projection [8]. The equation obtained by conducting the projection is as follows,

(3) $$\begin{array}{c} {\tilde{y}}_{p,lk}=\varphi_{k}^{H}y_{p,l}\\ \;\;\;\;\;\;\;\;\;\;\;\;\;=\sqrt{\tau_{p}\rho_{p}}h_{lk}+\sqrt{\tau_{p}\rho_{p}}\sum_{k^{\prime }\neq k}^{K}h_{lk}\varphi_{k}^{H}\varphi_{k^{\prime }}^{}\omega_{p,l}. \end{array}$$

As mentioned in Section 1, the MMSE estimator is adopted as the channel estimation technique. The MMSE estimator is given by

(4) $${\hat{h}}_{lk}=\frac{E\left\{{{\tilde{y}}^{*}}_{p,lk}h_{lk}\right\}}{E\left\{|{\tilde{y}}_{p,lk}{|}^{2}\right\}}{\tilde{y}}_{p,lk}=t_{lk}{\tilde{y}}_{p,lk},$$

where

(5) $$t_{lk}≜\frac{\sqrt{\tau_{p}\rho_{p}}\beta_{lk}}{\tau_{p}\rho_{p}\sum_{k^{\prime }=1}^{K}\beta_{lk}{\left|\varphi_{k}^{H}\varphi_{k^{\prime }}\right|}^{2}+1}.$$

### 2.2. Downlink Data Transmission

All APs treat the channel estimation in (4) and apply CB precoding to data signals to the users that are served. If AP *l* serves all users, then the transmitted signal from AP *l* is given by

(6) $$x_{l}=\sqrt{\rho_{d}}\sum_{k=1}^{K}\eta_{lk}^{1/2}{\hat{h}}_{lk}^{*}q_{k}.$$

The power constraint at each AP is given by

(7) $$E\left\{|x_{l}{|}^{2}\right\}\leq \rho_{d}.$$

Then power constraint (7) can be rewritten as follows,

(8) $$\sum_{k=1}^{K}\eta_{lk}\gamma_{lk}\leq 1,\;\;\;\;\mathrm{for}\;\;\mathrm{all}\;\;l,$$

where

(9) $$\gamma_{lk}≜E\left\{|{\hat{h}}_{lk}{|}^{2}\right\}=\sqrt{\tau_{p}\rho_{p}}\beta_{lk}t_{lk}.$$

When using the above formulas, the received signal at the user *k* is given by

(10) $$\begin{array}{c} r_{d,k}=\sum_{l=1}^{L}h_{lk}x_{l}+\omega_{d,k}\\ \;\;\;\;\;\;\;\;\;\;\;=\sqrt{\rho_{d}}\sum_{l=1}^{L}\sum_{k^{\prime }=1}^{K}\eta_{lk}^{1/2}h_{lk}{{\hat{h}}^{*}}_{lk}q_{k^{\prime }}+\omega_{d,k}. \end{array}$$

Then, $q_{k}$ is detected from $r_{d,k}$.

In this paper, the following spectral efficiency (12) as in [8] is used. Then, the obtained downlink spectral efficiency is given by (12). The (12) indicates the original cell-free massive MIMO downlink spectral efficiency.

Throughout this paper, $M_{k}$ is defined as a subset of APs serving user *k*. Then, α can be rewritten as follows,

(11) $$\alpha_{lk}=\left\{\begin{array}{c} I_{N}\;\;\;\;\;\;if\;l\in M_{k}\\ 0_{N}\;\;\;\;\;\;if\;l\notin M_{k} \end{array}\right..$$

Finally, the downlink spectral efficiency in the user-centric cell-free massive MIMO also can be described easily as (13) by applying the matrix α in (11).

### 2.3. Uplink Data Transmission

In the uplink data transmission phase, all *K* users send their data to the APs. The same channel condition is used in this phase as in the downlink data transmission phase. In this paper, we do not derive the uplink data transmission. The details for uplink data transmission are shown in [8].

## 3. Observation of Distribution for Per-User Throughput

**Figure 2:**
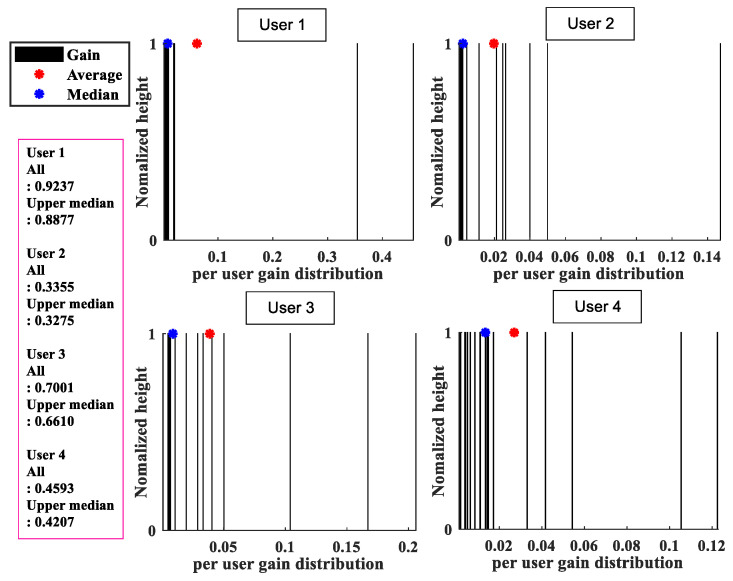
Distribution of per-user throughput for four users.

**Figure 3:**
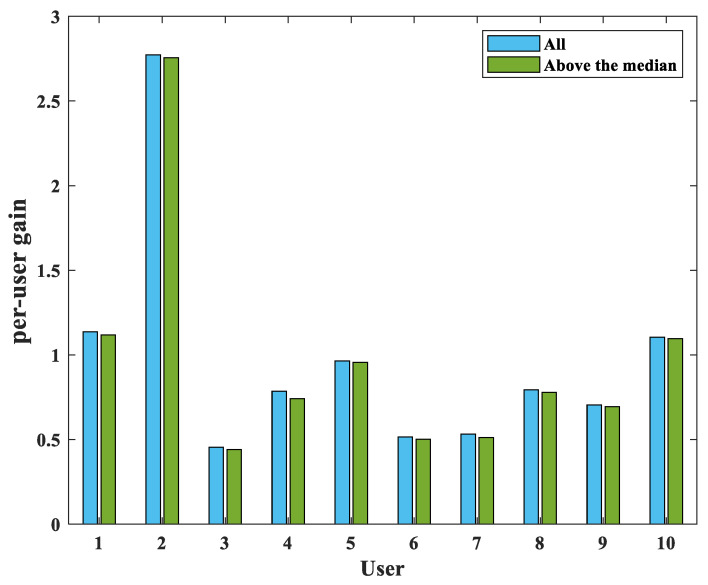
Bar graph of the total sum rate for 20 users and the sum of above the median.

The following per-user throughput distribution in the user-centric cell-free massive MIMO system is observed. Figure 2 shows the distribution of per-user throughput for four random users in a situation where 100 APs and 40 users exist. The most gains from each AP to specific user *k* are concentrated at a low value, and it can be seen that only a few APs occupy most of the total sum rate for users. That is, it can be seen from Figure 2 that each user can obtain very different gains from all connected APs. It is also found that the average of users’ throughput per user is significantly greater than the median, and as a result, a significant difference between these averages and the median more clearly shows the nonuniformity of the distribution. In Figure 3, the result of disconnecting the connection between the user and the AP based on the observation in Figure 2 is shown as a bar graph. It can be seen that the per-user throughput hardly changes even if only half of the number of APs serve a specific user *k*. Therefore, through the above observation and per-user gain, this paper proposes a scheme for serving unserved users in Section 4.

The proposed scheme is performed based on the above results, unlike the previous scheme, which is called MS assignment, in [20]. In the proposed scheme, since the number of users served by each AP is limited to a reasonable number (the number of installed antennas), for the feasible operation of the system, it is applicable even if the number of users in the network increases, and the amount of calculation does not increase in proportion to the number of users. In the following Section 4, an analysis of the previous scheme is conducted, and the new scheme is proposed.

## 4. Proposed Scheme and Power Control

In this section, the user-serving scheme for an unserved user is proposed. The proposed scheme in this paper is based on the observation and simulation result in Section 3, and this scheme supplements the limitations of the existing methods described in Section 4.1.

### 4.1. Analysis of the Previous Scheme

In this Section 4.1, according to the notation of the existing scheme [20], an unserved user is expressed as $k^{*}$, and an AP newly connected for the service of $k^{*}$ is expressed as $m^{*}$. The limitations of the previous scheme [20] are as follows:

(a) There is no consideration for the occurrence of another unserved user.
(b) It simply selects an AP that is close to the unserved user.

(12) $$R_{d,k}^{cf}=\log_{2}\left(1+\frac{N^{2}\rho_{d}{\left(\sum_{l=1}^{L}\eta_{lk}^{1/2}\gamma_{lk}\right)}^{2}}{N^{2}\rho_{d}\sum_{k^{\prime }\neq k}^{K}{\left(\sum_{l=1}^{L}\eta_{lk^{\prime }}^{1/2}\gamma_{lk^{\prime }}\frac{\beta_{lk}}{\beta_{lk^{\prime }}}\right)}^{2}|\varphi_{k^{\prime }}^{H}\varphi_{k}{|}^{2}+N\rho_{d}\sum_{k^{\prime }=1}^{K}\sum_{l=1}^{L}\eta_{lk^{\prime }}\gamma_{lk^{\prime }}\beta_{lk}+1}\right)$$

(13) $$R_{d,k}^{uc}=\log_{2}\left(1+\frac{N^{2}\rho_{d}{\left(\sum_{l=1}^{L}\eta_{lk}^{1/2}\gamma_{lk}\alpha_{lk}\right)}^{2}}{N^{2}\rho_{d}\sum_{k^{\prime }\neq k}^{K}{\left(\sum_{l=1}^{L}\eta_{lk^{\prime }}^{1/2}\gamma_{lk^{\prime }}\alpha_{lk}\frac{\beta_{lk}}{\beta_{lk^{\prime }}}\right)}^{2}|\varphi_{k^{\prime }}^{H}\varphi_{k}{|}^{2}+N\rho_{d}\sum_{k^{\prime }=1}^{K}\sum_{l=1}^{L}\eta_{lk^{\prime }}\gamma_{lk^{\prime }}\alpha_{lk}\beta_{lk}+1}\right)$$

The phenomenon that may occur due to a) is as follows. If the user $k^{*}$ of the existing scheme [20] is served only by the AP $m^{*}$, a new unserved user occurs. This causes a problem whereby, in a situation where the number of antennas of the AP is not large, the probability of generating new unserved users increases as the number of users increases. In other words, it is judged that this is an unreasonable service provision method, because the service to existing users is not guaranteed when a new connection is created.

In case (b), even if there are many APs serving user $k^{*}$, there is a problem in that AP $m^{*}$ may occupy a fairly high percentage of the sum throughput of user $k^{*}$. This can be inferred from the distribution in Figure 2 of Section 3. If a connection with a new user is created without considering this non-uniformity of throughput, the throughput of other users that are already receiving the service will be greatly reduced. Consequently, it causes a situation in which the average throughput of the entire network is lowered, which leads to the deterioration of the system performance.

### 4.2. Proposed User Serving Scheme

To solve the above problems, the following new scheme is proposed. The newly proposed scheme works when unserved users exist, and this scheme can serve users within the area, while solving the limitations in (a) and (b).

Algorithm 1 describes an efficient user service scheme that can serve the unserved users when a large number of users exist within the area. First, the limitation (a) can be solved through STEP 1. It is confirmed that the case in which the previously served users are dropped in order to serve unserved users is prevented. Once the set P is selected through STEP 1, the set S is defined as the set of users served by the set P. In STEP 2 and STEP 3, the indices and gain are extracted. The limitation in (b) can be solved by first performing disconnection of the lower-level APs connected to the user supported by the most APs. In STEP 4, the priority is given in order of receiving service from the largest number of APs among users in set S. Through this, it is possible not to damage the gain of existing users served by a relatively small number of APs as much as possible. In STEP 5, the index of the APs that serve the gains less than the median of the total gain is extracted for each user in the set S according to the priority given in STEP 4. Finally, in STEP 6, a connection with user *i* is made according to the priority given in STEP 5, and the signal-to-interference-plus-noise-ratio (SINR) accumulation for user *i* is performed. If the cumulative sum of the SINR for user *i* is larger than or equal to the threshold value ξ, STEP 6 stops, and the search for other unserved users is performed again.
**Algorithm 1** Proposed Unserved user Serving Scheme$\mathrm{Input}:\;\mathrm{Channel}\;\mathrm{Matrix}:\;H,\;\mathrm{Subset}\;\mathrm{of}\;\mathrm{AP}\;\mathrm{for}\;\mathrm{user}\;k:\;M_{k},\;\mathrm{Channel}\;\mathrm{Estimation}:\;\gamma ,$                  ConnectionMatrix:α,Threshold:ξ    **for i**=1→*K*    **if $M_{i}$** =⌀        **STEP 1:** Select P candidate APs with high channel gain γ from user *i*.        (If there is a user served only by a specific AP among users served by the APs in P,        the corresponding AP is excluded from group P.)        **STEP 2:** Define the set of users served by P as S.        **STEP 3:** Extract the index set and channel gain of users in S.        **STEP 4:** Prioritize and list them in the order in which they are served by the largest      number of APs among users in S.        **STEP 5:** Extract APs that provide the gains below the median in descending order      for each user value from among the gains listed in descending order      for each user.        **STEP 6:** Connect the user *i* with the AP sets extracted in **STEP 5** and execute      the SINR cumulative sum (δ).        **while** ξ>δ    The APs of the set extracted in STEP 5 are    sequentially connected to the user *i*.        **end while****end if****end for**

In the following Section 5, the performance for the newly proposed scheme is applied and is compared with previous scheme. Comparisons with three cases are performed to confirm that the proposed scheme works well in the user-centric cell-free massive MIMO model.

### 4.3. Computational Complexity

The proposed scheme has the following complexity. The number of unserved users existing before STEP 1 is denoted by U, and I is the count of iterative cumulative sums for the threshold value ξ in STEP 6. As a result of STEPs 1–5, if the number of APs newly connected to a specific unserved user $k^{*}$ is A, the number of other users served by APs in a set A (excluding unserved user $k^{*}$) can be denoted by S. Then the complexity of the proposed scheme can be calculated as O(UI(5A+2S+1)). Naturally, since the number of unserved users in the area increases in proportion to the total number of users, if the number of unserved users in the system is small, the complexity of the scheme may very low.

### 4.4. Power Control and Pilot Assignment

To clearly confirm the performance of the proposed algorithm, this paper adopts an equivalent gain power control algorithm as in [8,25] instead of an additional power control algorithm. In the user-centric cell-free massive MIMO model, the power control coefficient between AP *l* and user *k* can be written by

(14) $$\eta_{lk}=\eta_{lk}={\left(\sum_{k^{\prime }=1}^{K}\alpha_{lk^{\prime }}\gamma_{lk^{\prime }}\right)}^{-1}.$$

When the number of users is very large, the use of duplicate sequences between users is inevitable. Therefore, in such a situation, a proper method for allocating pilots efficiently is required as [8].

## 5. Simulation Results

In this Section 5, the proposed scheme is compared with the existing user-serving scheme in the user-centric cell-free massive MIMO model and the original cell-free model. For all simulations, it is assumed that APs serve as many users as the number of installed antennas and the length of the orthogonal pilot sequence is equal to the number of users. Table 2 shows the simulation parameters used in this paper.

### Simulation Analysis

**Figure 4:**
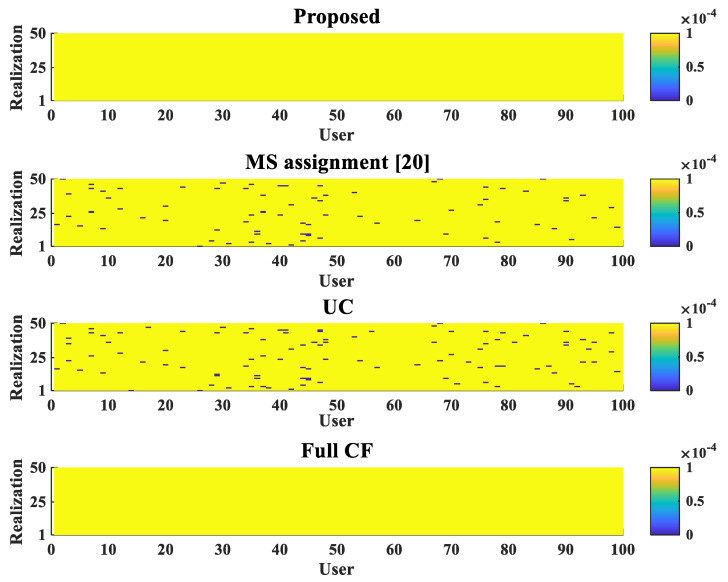
Realization of unserved-user for four cases.

Figure 4 shows the number of unserved users in the proposed scheme and other cases. The simulation was performed through a total of 50 realizations, and both the number of APs and the number of users were set to 100. It is shown that unserved users did not occur in the given environment when the proposed scheme was applied. In this figure, UC indicates a user-centric scheme that does not apply any user-serving scheme. When no schemes were applied, it can be seen that the number of unserved users was very high. Furthermore, it was somewhat relaxed when the scheme in [20] was applied, but it was confirmed that there were still some existing unserved users. It is also noteworthy that when the scheme in [20] was applied compared to the case of UC, although the existing unserved users disappeared, the connection with the existing users was disconnected, and new unserved users were generated. However, the unserved users disappeared when the proposed scheme was applied; therefore, it can be seen that the proposed scheme works properly.

**Figure 5:**
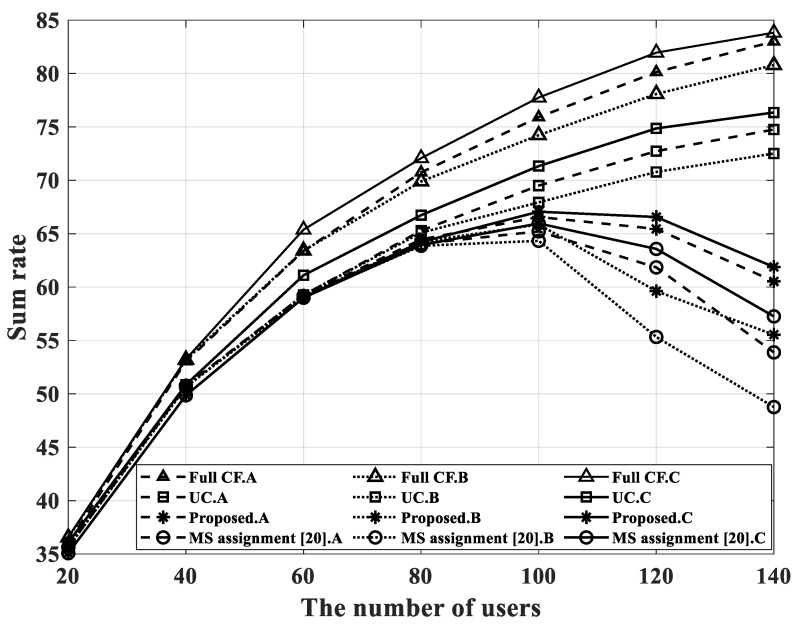
Sum rate performance for four cases.

The simulation in Figure 5 shows the sum rate for each case in three different situations, and it was also implemented in an environment with 100 APs. There were three different situations as follows:

- Imperfect CSI and orthogonal pilot per user.
- Imperfect CSI and only 60 orthogonal pilot sequences in the system.
- Perfect CSI and orthogonal pilot per user.

If there were more than 60 users in the situation of B, the pilot allocation method in [8] was applied, and the imperfect CSI environment was implemented as the MMSE estimator in Section 2.1. As can be seen from Figure 5, when the user-serving schemes were applied, the total sum rate increased at the beginning as the number of users increased; then, it decreased after 100 users due to the low-rank problem in all situations from A to C. Generally, in the cell-free massive MIMO network model, only a small number of antennas are installed in each AP and performance degradation is inevitable when the number of users increases. However, it is confirmed that the decrease rate in the proposed scheme was smaller than the scheme in [20]; moreover, the proposed scheme achieve a higher sum rate than the previous scheme in all situations. The proposed scheme serves new users while maintaining the connection with existing users.

**Figure 6:**
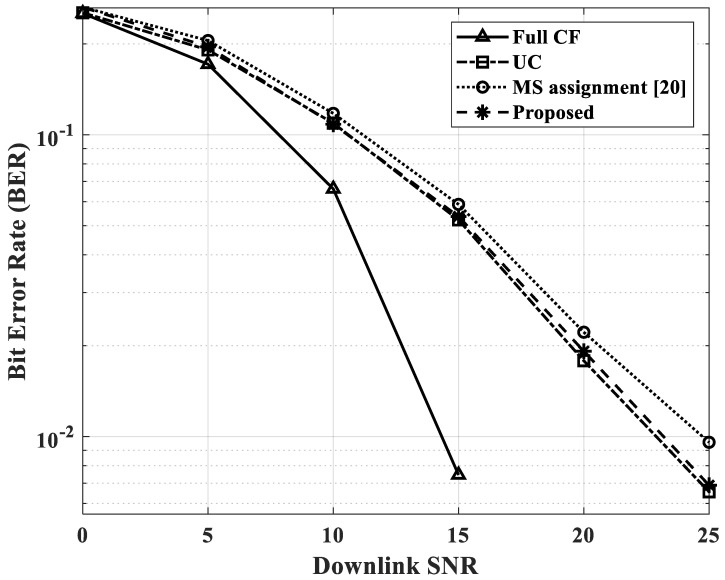
BER performance for four cases.

The performance of bit error rate (BER) for the four cases is shown in Figure 6. In this simulation, the number of APs and users was set to *L* = *K* = 100. Since the number of users was 100, the unserved users were likely to occur in other cases except for Full CF. In this paper, we assumed that the BER of the unserved users was 0.5 to obtain effective BER performance. It is confirmed that when the proposed scheme was used, the number of unserved users was greatly reduced compared to UC, and at the same time, a slightly improved BER performance was obtained compared to the scheme of [20]. Since CB (Conjugate Beamforming) was applied to the precoding algorithm, and all four cases corresponded to a very dense environment in which the number of users was set equal to the number of APs, the overall BER performance in the simulation shown in Figure 6 was rather poor.

**Figure 7:**
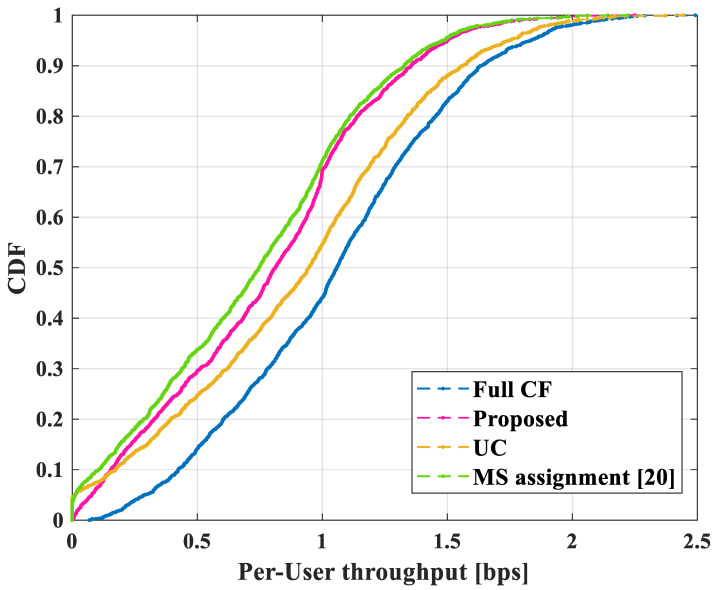
CDF of per-user throughput for four cases with two antennas.

The CDF performance for four cases is shown in Figure 7. In this simulation, two antennas were installed in each AP, and it was assumed that there were *L* = 100, *K* = 60 in the area. In order to perform in a real environment and reduce computational complexity, the equal gain algorithm in [8,25] was applied as a power control algorithm. Among all cases, the CDF performance of the original cell-free massive MIMO (Full CF) outperformed the other cases. This is because there is no limit on the number of users that can be served for each AP; then, the Full CF mode can serve all users in the area. This characteristic of a cell-free model makes the absolute value of the data rate serving per-user large. It is also shown that the proposed user-serving scheme ensures service for the worst-10% of users compared with the other cases except for the Full CF case. The performance of per-user throughput in the bottom of 70 percentage of users is also improved by the proposed scheme.

**Figure 8:**
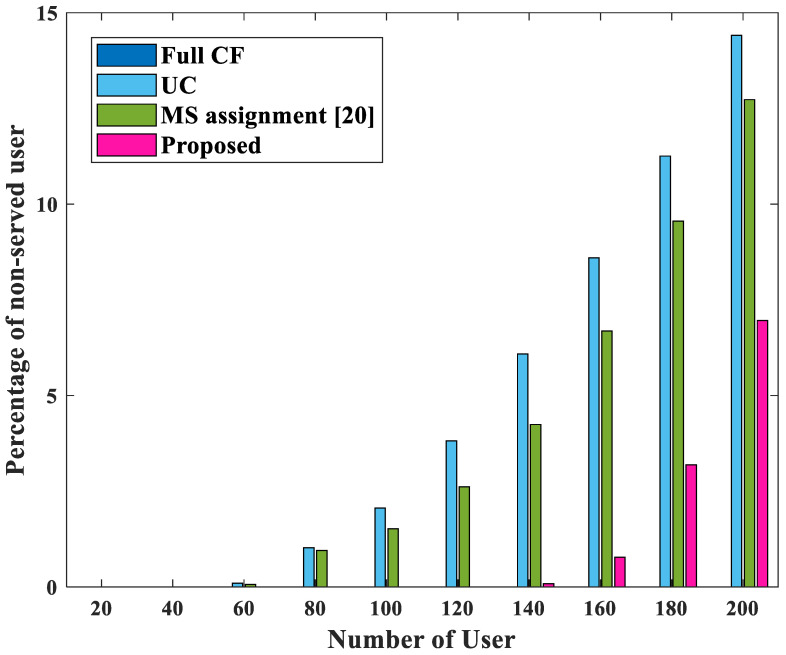
The number of unserved users for four cases.

Finally, Figure 8 shows the ratio of unserved users according to the number of users. This simulation considers the P as $\frac{1}{2}L$, and *L* = 100. In the case of the original cell-free massive MIMO, unserved users do not occur because of its property, but the other three schemes have unserved users. In the proposed scheme, if there were more than 140 users, unserved users occured. This is because the candidate AP set in P does not include all APs to maintain reasonable computational complexity in the proposed scheme. In addition, in a situation that has a very large number of users, the users served by only one AP should exist in the set P. This means that as the number of users within an area increases, the number of users served by only one AP also increases. This case is excluded from STEP 1 of the proposed scheme. In the proposed scheme, since the service of existing users should be given priority, even if the proposed scheme is used, it is natural that unserved users would exist in the simulation of Figure 8.

## 6. Conclusions

In this paper, a user-serving scheme was proposed to serve all users while satisfying realistic fronthaul requirements and ensuring a connection for existing users when a large number of users exist in a user-centric cell-free massive MIMO system. To reduce the complexity and the burden on the fronthaul, this paper adopted conjugate beamforming (CB) for the precoding algorithm and limited the number of antennas (equal to the number of serving users). It was confirmed through several simulations in Section 5 that when the proposed scheme was applied, the network system could serve a very large number of users in a feasible way. In addition, it is noteworthy that the sum rate performance of the proposed scheme in several situations was improved compared to the existing method. Through the proposed scheme, the spectral efficiency and per-user throughput performance were improved while maintaining the connection with the existing users. In particular, it is shown that all users can receive service as in the full cell-free massive MIMO model, ensuring service for the bottom 10% of users. It was shown that the proposed scheme can efficiently serve the unserved users in the user-centric cell-free massive MIMO system even when the number of users in the area is very large.

## Figures and Tables

**Table 1:** Notations and Symbols.

Notation	Definition
${\left(\cdot \right)}^{T},{\left(\cdot \right)}^{H}$	Transpose and conjugate transpose
$E\left\{\cdot \right\}$	Expectation value
${\left(\cdot \right)}^{*}$	The complex conjugate operation
$\left||\cdot \right|{|}_{F}$	Frobenius norms
$N_{C}\left(\cdot ,\cdot \right)$	Circularly symmetric complex Gaussian distribution

**Table 2:** Simulation Parameters and Schemes.

Parameters	Value
Area	1 × 1 k$m^{2}$
Number of antennas for AP	2 or 4 antennas
Number of antenna for users	Single antenna
User antenna height	1.65 m
$\rho_{p},\rho_{d}$	100 mW, 200 mW
Length of the pilot sequence	The number of users or 60
Modulation	QPSK
Bandwidth	20 MB
Noise figure	9 dB

## Data Availability

Not applicable.
